# Perfect Anæsthesia

**Published:** 1865

**Authors:** 


Perfect Anæsthesia.—A Nevada paper mentions that a Dr. Ross, being a merciful man, was about to clip the ears of a fine-blooded rat-terrier. He accordingly procured some chloroform, and after administering several doses by means of a sponge, succeeded in producing a satisfactory state of insensibility. He clipped the pup's ears beautifully, and wired them up in splendid style. So far the operation was a success, but when he came to wake up his patient, the juvenile canine was dead.

